# Designing cultural multilevel selection research for sustainability science

**DOI:** 10.1007/s11625-017-0509-2

**Published:** 2017-11-21

**Authors:** Michelle A. Kline, Timothy M. Waring, Jonathan Salerno

**Affiliations:** 1 Department of Psychology, Simon Fraser University, Burnaby, BC Canada; 2 Institute of Human Origins, Arizona State University, Tempe, USA; 3 School of Human Evolution and Social Change, Arizona State University, Tempe, USA; 4 School of Economics, University of Maine, Orono, USA; 5 Mitchell Center for Sustainability Solutions, University of Maine, Orono, USA; 6 Environmental Studies Program, University of Colorado Boulder, Boulder, USA

**Keywords:** Cultural evolution, Group-level cultural selection, Social dilemmas, Cultural multilevel selection, Evolution of sustainable systems

## Abstract

Humans stand out among animals in that we cooperate in large groups to exploit natural resources, and accumulate resource exploitation techniques across generations via cultural learning. This uniquely human form of adaptability is in large part to blame for the global sustainability crisis. This paper builds on cultural evolutionary theory to conceptualize and study environmental resource use and overexploitation. Human social learning and cooperation, particularly regarding social dilemmas, result in both sustainability crises and solutions. Examples include the collapse of global fisheries, and multilateral agreements to halt ozone depletion. We propose an explicitly evolutionary approach to study how crises and solutions may emerge, persist, or disappear. We first present a brief primer on cultural evolution to define group-level cultural adaptations for resource use. This includes criteria for identifying where group-level cultural adaptations may exist, and if a cultural evolutionary approach can be implemented in studying a given system. We then outline a step-by-step process for designing a study of group-level cultural adaptation, including the major methodological considerations that researchers should address in study design, such as tradeoffs between validity and control, issues of time scale, and the value of both qualitative and quantitative data and analysis. We discuss how to evaluate multiple types of evidence synthetically, including historical accounts, new and existing data sets, case studies, and simulations. The electronic supplement provides a tutorial and simple computer code in the R environment to lead users from theory to data to an illustration of an empirical test for group-level adaptations in sustainability research.

**Electronic supplementary material:**

The online version of this article (10.1007/s11625-017-0509-2) contains supplementary material, which is available to authorized users.

## Introduction

The global sustainability crisis is a result of a uniquely human form of adaptability. Like other animals, humans consume natural resources to survive and reproduce. However, humans stand out among animals, in that we cooperate in very large groups to exploit natural resources (Gowdy and Krall 2013), and we accumulate survival and resource exploitation techniques across generations via cultural learning (Richerson and Boyd 2005). This unique set of capacities has permitted human populations to expand to every major terrestrial ecosystem (Henrich 2015). Our material and energetic consumption and our impacts on earth’s systems are so severe that a new geological epoch, the Anthropocene, has been proposed for the current period of human-dominated earth-system processes (Steffen et al. 2007). Thus, the global sustainability crisis is a direct result of human cultural and cognitive adaptability.

Unfortunately, the short-term ecological successes created by human adaptability are now putting the longer-term survival of human populations at risk. Critical resources are being exhausted. As these resources disappear, shortages can reveal underlying environmental social dilemmas—conflicts between resource users in which the best option for individuals differs from the best option for the group as a whole. For example, an individual may benefit most from unlimited fishing on a commonly owned reef, while the best option for the village as a whole may be lower rates of fishing that maintain the reef fishery in the long-term. Environmental social dilemmas like this are embedded in many of our most vexing sustainability challenges, from overfishing to carbon dioxide emissions. Unresolved environmental social dilemmas lead to sustainability failures, including resource depletion, stock collapse, over-pollution, and biodiversity loss.

However, human adaptability can also work in the other direction. The dynamics of culture and cooperation play a central role in the emergence and maintenance of sustainable social–ecological systems. Social dilemmas are sometimes resolved through cooperation among group members, and social learning between groups can spread innovative sustainability solutions. The creation, emergence, and spread of these solutions can be described in terms of cultural evolutionary processes, at multiple group and individual (or organismic) levels (multilevel selection; Waring et al. 2017). In this view, environmental cooperation, sustainable behaviors, and institutions may evolve among human groups via cultural adaptation for environmental management.

Although the cultural evolution of sustainable behaviors and institutions has been demonstrated in theory (Waring et al. 2017), empirical research is needed to explore and test the implications of multilevel selection theory for sustainability science. In the present paper, we build on Waring et al.’s (2015) framework to provide a guide for empirical research on the cultural evolution of social–ecological systems. First, we explain the practical advantages of an explicit evolutionary approach (Sect. 2). Next, we outline how group-cultural adaptations for environmental management evolve, and provide a roadmap for identifying them (Sect. 3). Building on this basic framework, we discuss practical steps for evolutionary study design (Sect. 4), highlight major concerns for data collection that are particularly relevant for future studies (Sect. 5), and offer analytical tools for cases with sufficient quantitative data (Sect. 6).

## Why an explicit evolutionary approach is needed

We aim to advance sustainability science by making evolutionary analyses more practicably applicable in research. Culture and evolutionary processes are already important to sustainability research in general: Ostrom (2008) suggests that institutions for collective action do evolve, but does not specify a mechanism. Gual and Norgaard (2010) use a generalized evolutionary framework for studying social–environmental interactions, and Beddoe et al. (2009) assert that evolutionary principles should be at the center of redesigning worldviews, institutions, and technology. We agree that cultural dynamics are embedded in institutional change (Caldas et al. 2015; Kinzig et al. 2013), and that cultural evolution should therefore be utilized in efforts to address global sustainability challenges (Ehrlich, 2009). However, there is an implementation gap. Explicit arguments about the ways in which cultural evolution is relevant to sustainability are rare (but see Richerson et al. 2002), and there are still no guidelines to make cultural evolutionary theory directly applicable to sustainability research. In this paper, we focus on closing that gap to facilitate research at the nexus of cultural evolutionary theory and sustainability science. We aim to show researchers how to specify what is evolving, how it evolves, and what we can learn by studying its evolution.

A thorough primer on cultural evolutionary theory is beyond the scope of this paper, so we introduce core terms that are crucial to understanding how cultural evolution can be used to study social dilemmas, and the role this can play in sustainability science (see Table 1). For accessible and thorough reviews of cultural evolutionary theory and empirical evidence, see Richerson and Boyd (2005) and Henrich (2015). For our current purposes, it is sufficient to say that cultural evolution is concerned with the evolution of cultural traits which may be observable at either the individual level (e.g., behaviors and beliefs), or at the group level. Group-level cultural traits can include cultural phenomena that do not exist at the individual level, including institutions, laws, and policies as well as distributions of traits (Smaldino 2014). For example, the percentage of membership in institutions, the frequency of law violations per capita, or degree of enthusiasm for a policy are all group-cultural traits because they are properties of groups, rather than individuals.

**Table 1:** Itemizes and defines key terms in cultural evolution

Term	Definition
Cultural trait	A general term for a unit of culture for analysis. Traits can exist at the individual or group levels. Traits can be discrete, such as individual beliefs or behaviors, or group-level norms or institutions. Traits can also be distributions or frequencies which exist exclusively at the level of groups
Cultural transmission mechanisms	Cultural transmission mechanisms are the routes through which cultural traits are learned and spread. Cultural transmission can create forces of cultural evolution that run counter to natural selection. Transmission mechanisms may be content-based, responding to characteristics of the cultural trait itself (such as memorability), or context-based, responding to external factors associated with the trait, such as who is displaying the trait (model-based) or how common the trait is (frequency-based)
Cultural selection	Cultural selection is any process that causes a filtering of cultural traits in a population, including both natural selection and the selective effects of cultural transmission on cultural traits
Natural selection	The proliferation of traits in a population due to greater survival or reproduction of their hosts. Natural selection’s effect on some cultural traits may be weak, where the trait’s spread is not closely coupled with the survival and reproduction of its host
Cultural adaptation	A cultural adaptation is a cultural trait with functional utility deriving from cultural selection. Cultural adaptations can evolve at individual or group levels. However, learning mechanisms can also lead to afunctional (neutral) or dysfunctional (maladaptive) traits, which are not cultural adaptations by this definition
Supporting behaviors/institutions	Behaviors and institutions that enable, stabilize, or reinforce some cultural trait, such as a cooperation regime or institutional feature. Note: support is often context-dependent and may coevolve with a focal trait

For a more detailed review of terminology, see Mesoudi (2011). For a review of the theoretical basis of categories in this table, see Henrich and McElreath (2003)

To comprehend how groups and cultural evolution can play a role in social dilemmas, we must contrast cultural evolution with genetic evolution. Evolution is a general term for change over time, but when we use the term “cultural evolution” we mean this in a more specific sense, covering the changing frequencies of cultural traits at the population level, just as genetical evolution tracks the changing frequencies of genes in a gene pool. In both domains of evolution, natural selection is the proliferation of traits in a population due to greater survival or reproduction of their host organisms. Cultural evolution does differ from genetic evolution in two key ways: First, while an individual’s genetic makeup is fixed, their cultural makeup changes across their life span. Second, cultural traits can be copied from any number of cultural peers, younger and older, and can be substantially edited by individuals during their lifetimes. Culture can also spread through several different transmission mechanisms. In contrast, genes are inherited from only two parents. As a result, cultural traits can reproduce and disappear in a population through cultural transmission mechanisms, as well as through natural selection. For example, a trait may spread because of its content: including whether it is easier to learn (as with singing a catchy tune), or fits in with pre-existing learning biases (as with a scary story). In addition, a trait can spread because of its context, including whether it is associated with a successful or prestigious person (as with green marketing campaigns), and depending on its relative frequency (as with norms regarding recycling).

The variety of ways through which cultural traits can spread or disappear makes cultural evolutionary explanations at once complex, powerful, and necessary for a full account of human behavior. It also makes cultural evolution a prime candidate for theoretical explanations of seemingly maladaptive behavior, including altruism among non-kin, and group-level cooperation, such as individually costly solutions to social dilemmas. These types of behaviors evolve in cultural systems because the transmission of cultural traits is decoupled from the genetic reproduction of their hosts. Whether a cultural trait can spread or not, it improves its hosts’ genetic fitness and cultural traits can also persist and spread when they cause a group to expand, in competition with other groups (Richerson et al. 2016). This is called group-level cultural selection (or cultural group selection^1^). Crucially, these benefits may apply in different degrees to individual members in the group, and need not benefit individuals directly. This makes group-level cultural selection a particularly useful tool for addressing social dilemmas, since in a social dilemma, by definition, group benefits always come at a cost to individuals.

In this paper, we show how cultural evolutionary theory can improve sustainability research by helping to solve social dilemmas over environmental resources. To make the benefits of this evolutionary approach concrete, we will sometimes refer to an example of sustainability certifications. Sustainability certifications of consumer products, such as coffee, fruits and vegetables, timber, and seafood, are an attempt to facilitate sustainable production practices by creating benefits for environmentally and/or socially beneficial practices, usually by fetching a higher price for sustainability-labeled goods. Certification is only possible when producers adopt sustainable practices, which is costly to individual producers, in that they increase operating costs, decrease short-term production efficiency, and may require up-front investment to change from existing practices. Consider, for example, the social dilemma facing producers in a certification context. Individual producers can maximize short-term benefits by harvesting the largest possible amount for the lowest possible cost (e.g., clearing primary forest to plant additional coffee or oil palm trees, or using prohibited pesticides). Certifications, however, impose criteria that prohibit some of these options, preventing producers from maximizing their short-term benefits in this manner. At the same time, these constraints maximize benefits for at least one broader group: providing higher quality product for consumers, preventing the contamination of local community resources through pollution, and protecting public goods, such as clean air and water.

What makes this a social dilemma is that, from an individual producer’s perspective, their portion of these group benefits does not compensate for their individual costs. In practical terms, this means that producers who unilaterally pursue the costly option of sustainable production might go out of business. In cultural evolutionary terms, the cultural trait of sustainable production is selected against because producers who practice it would be eliminated from the population. In theory, certifications can solve this problem by creating benefits that flow back to individual producers, for example, through marketing of certified products that carry a label or guarantee and fetch higher prices. This allows individual producers to offset the costs of participation (i.e., following the rules of the certification). Importantly, the certification mechanism must function at the group level, providing group-based enforcement and monitoring, to ensure that producers do not revert to short-term strategies. In practice, even individuals in a certified system may still attempt to maximize production and reduce the long-term benefits to the group. The overall outcomes are, therefore, the result of a balance or tradeoffs between individual and group interests. Throughout this paper, we will use examples of this sort to explore the possibilities for cultural evolutionary theory to address social dilemmas in sustainability science. It is notable that, in practice, certifications encounter all the challenges inherent in cooperative dilemmas.

## How group-cultural adaptations for environmental use evolve

Group-cultural adaptations are diverse and multifaceted (Richerson et al. 2016; Smaldino 2014), but identifying them requires careful attention to the processes of cultural evolution. For example, group-beneficial behaviors or institutions are often assumed to be group-level cultural adaptations. This is a mistake, because a group-beneficial trait may be adaptive or advantageous, but may not have spread through the process of cultural selection. This falls outside the strict definition we embrace here: group-level cultural adaptations are group-beneficial traits which emerged via group-level cultural selection. In this section, we briefly explain how group-cultural adaptations evolve, and thereby how to identify both the process of group-level cultural selection, as well as its outputs.

We focus on social dilemmas in this paper for two reasons. First, social dilemmas throw the interests of individuals into conflict with those of groups. As a result, selection on individuals in social dilemmas does not favor group-functional traits. This makes group-level adaptations in response to social dilemmas rare, but easier to detect, and harder to explain without group-level selection. This is a strong test of our framework because if it fails here, it is unlikely to succeed elsewhere. In addition, we focus on social dilemmas because they feature centrally in many problems of environmental sustainability, and provide a clearly demarcated target of study for researchers in that field.

**Fig. 1:**
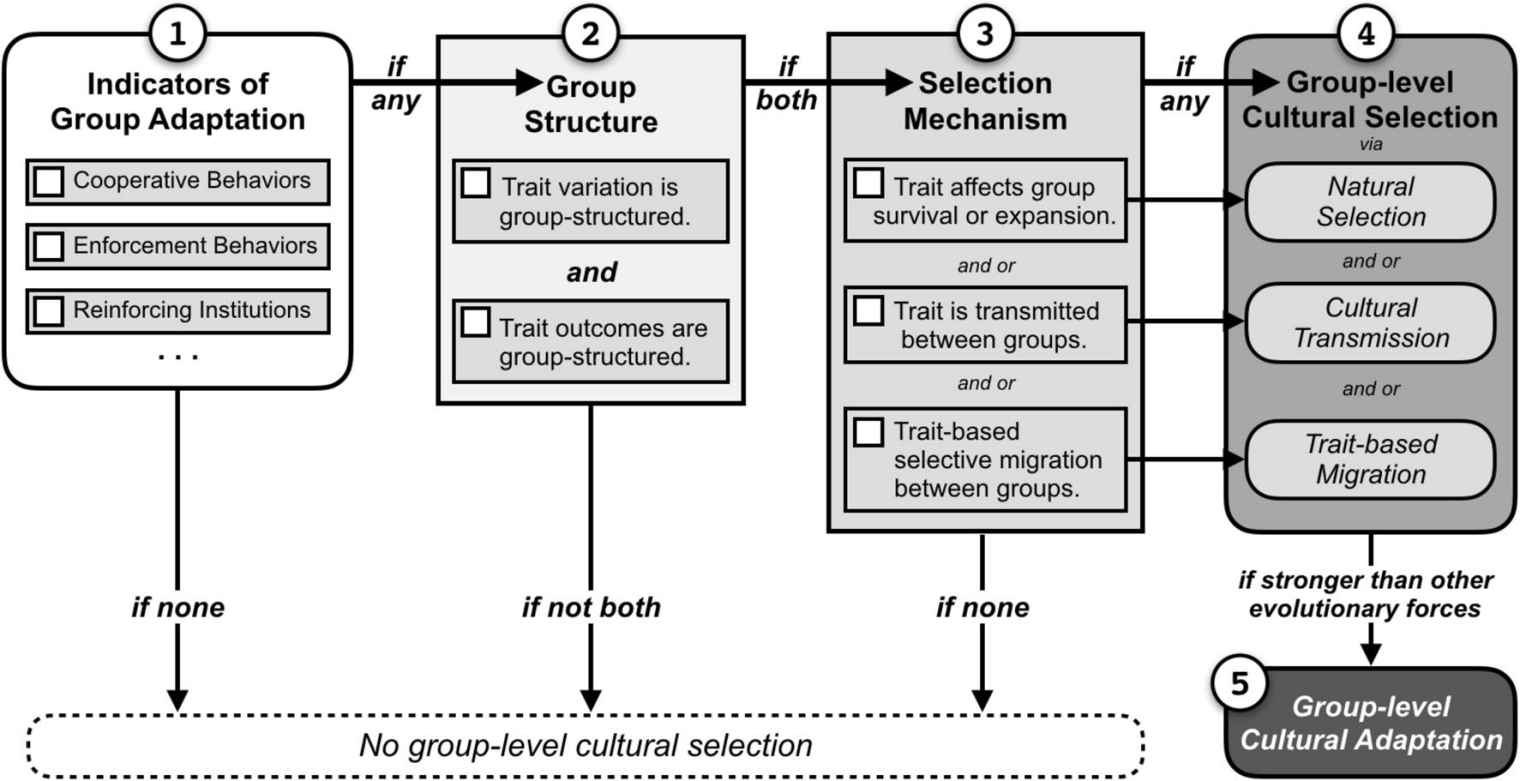
A flow chart for constructing evolutionary hypotheses about the role group-level cultural selection in the evolution of a trait under study. (1) Group-functional behaviors provide indication that the focal trait may be a group-level cultural adaptation. Boxes 2–3 summarize absolute requirements for positively identifying the role for group-level cultural selection: (2) the trait must to be group-structured, and must influence group outcomes and (3) the trait must spread via one or more mechanisms of cultural selection. If these conditions are met (4) group-structured cultural selection (Zefferman and Mathew 2015) is occurring and, if stronger than other evolutionary forces, can result in (5) group-level cultural adaptation

The most useful indicators of group-level adaptations include factors that are unlikely to emerge via individual-level selection alone, such as cooperation, and behaviors or institutions that organize or reinforce cooperation (Fig. 1, Box 1). Some of the most widely applicable and best-researched indicators of potential group-level cultural adaptations for environmental use and management are Ostrom’s (1990) institutional design principles (see also Dietz et al. 2003; Anderies et al. 2004; Wilson et al. 2013). Ostrom’s institutional principles provide an intuitive guide for identifying indicators of group-cultural adaptation (Wilson et al. 2013). Thus, the more group-functional indicators present, the more convinced we may be that group-level cultural selection was at play, and the greater the potential for a dedicated evolutionary study. As noted above, processes of social self-organization, such as group-like assortment of cooperators (Nowak 2006), can also give rise to group-beneficial traits. This is why group-beneficial traits alone are not sufficient evidence of group-level cultural adaptation.

For group trait to evolve via group-level cultural selection, it must be group-structured, it must have group-structured outcomes (Fig. 1, Box 2), and it must spread via a mechanism of cultural selection as a result of those group benefits (Box 3). Traits are more likely to experience group-level selection when they vary more between groups, relative to within groups (see Sect. 6 for quantitative methods). In addition, the group-level trait must also influence group-level outcomes. Finally, for a trait to be a cultural adaptation, it must spread between groups via some mechanism of cultural selection (Fig. 1, Box 3). Researchers can use this flow chart to evaluate whether group-level cultural selection may be important in their study system, and (if yes) to construct hypotheses about whether a group-level cultural adaptation may be the result.

To result in a group-level adaptation, group selection must also be stronger than individual selection and evolutionary forces, such as drift and mutation (see Sect. 6 for methods to determine strength of selection, across levels). This is a particular concern for social dilemmas, where individual-level and group-level cultural selections are in opposition; weak group-level cultural selection may also be swamped by other evolutionary forces, such as drift. For example, individual selection is apparently prevailing in the social dilemma of water conservation in California. Overconsumption of fresh water continues since some individuals persist in watering their lawns because they can afford to pay a high water bill in the midst of a decades-long drought. Alternatively, when group-level selection is stronger than individual-level selection, group-beneficial practices may emerge at the expense of individuals’ direct interests. For example, this would describe hunters who forgo shooting female animals—which costs them in terms of fewer kills and less meat for their efforts—but which benefits the broader group of hunters because it promotes a faster rate of reproduction by the prey species. This kind of group-beneficial trait requires a high degree of cooperation within the group, because cheaters could take advantage of the benefit without paying the cost, and eventually out-compete the cooperators.

Once it has been established that group-level cultural selection is at play, the next step is to determine how the various mechanisms (Box 3) operate, possibly in conjunction. Natural selection builds most directly on the general idea of competition among groups. A group-structured cultural trait is under positive natural selection when it promotes the survival, growth, and reproductive success of the group that adopted it. In this case, the trait promotes its own survival and replication. Group-level genetic selection is thought to be nearly non-existent in other species and rare among human groups, because human genetic variation does not have strong “group structure”, but human cultural variation does (Bell et al. 2009). In group-level cultural selection, survival and reproduction can be a function of economic gain at the group level, caused by the beneficial effects of the cultural trait.

The second mechanism of spread, cultural transmission, itself consists of a handful of transmission mechanisms, all of which rely on social learning as a route to the replication of cultural traits. It is unique to cultural evolution, though its dynamics are similar to contagion: social learning allows for the transmission of strategies and behavior not strictly linked to natural selection. Thus, traits can spread through populations without respect to the host’s well-being like a virus (Henrich et al. 2008). At the individual level, transmission happens through a variety of modes of learning, including observation, copying, learning via cultural artifacts like books or recordings, and learning from others (Kline 2015). Individuals can “copy” other individuals in successful groups in such a way that a group-structured trait can pass between groups. This may occur with functional traits, such as bow-and-arrow technology or with apparently useless ones like hairstyle trends. If traits are not only group-structured but also affect a group’s outcomes, such as norms of in-group cooperation, then cultural transmission works as a mechanism of group-level cultural selection.

Group-level trait transmission through social learning is built on individual human learning capacities and can be facilitated or impeded by group organization, including leadership. For example, the industry-level copying of sustainable labeling is common, including both the proliferation of third-party certified products, as well as the spread of “natural” and “sustainable” claims on product labels, also known as “greenwashing”. Features of a trait itself can also potentiate its spread: traits that are easier to observe, easier to learn, or associated with success are more likely to proliferate—particularly across group boundaries where face-to-face contact may be minimal. For example, technologies and tools whose uses are immediately obvious (e.g., a blade with a handle) spread more quickly than more practices with opaque benefits (e.g., boiling water or washing hands). Despite the health benefits of disinfected water, the role of boiling is difficult to understand, making these practices slow to spread (Rogers 1990).

A third major mechanism of spread is expansion through trait-based migration (Rogers 1990). The movement of ideas, beliefs, and behaviors can occur when people move, sometimes resulting in trait transmission in the emigrant’s new home, and changes in trait frequencies as well as trait outcomes in the group (Boyd and Richerson 2009; Henrich 2004). Generally, group-level cultural traits that encourage emigration will result in reduced group membership, while traits that encourage immigration will result in increased group membership. This mechanism may apply whenever individuals have freedom of movement to the degree that they can “vote with their feet” (Boyd and Richerson 2009). Migration can also influence the distribution of cultural traits, if it changes the overall composition of the group. If this change is large enough to affect the group-level structure of the trait, it may influence selection at sub-group or individual levels. And, on a broad scale, migration can sometimes be a direct mechanism of selection if groups disperse, relocate, or dissolve entirely. For example, a lack of local opportunity may sometimes cause college students who have obtained an environmental education to migrate from their home state to find greater work opportunities elsewhere. This “brain drain” would quickly swell the proportion of environmental workers in one region, while decreasing their presence in the other. This could create a cycle whereby sustainability practices become increasingly common and efficient in one region, and less common or well-executed in another.

Mechanisms of group-level cultural selection may often operate in tandem. Natural selection and cultural transmission are observable where sustainability certification systems connect producers and consumers in an economically relevant group structure. Natural selection acts when an agricultural producer minimizes costs using the cheapest and most effective pesticide, and accumulates savings as profit. Where profit determines a producer’s success, producers with the efficient pesticide trait will out-compete those that use more expensive but environmentally safer methods; cheap-but-toxic pest control will become common among competing producers. In the absence of government regulations limiting harmful pesticide use, effective certifications mandate certain producer practices (e.g., prohibit harmful inputs and prevent cheating) to which consumers assign value. Certifications accomplish this by shifting the level of cultural selection (see Waring et al. 2015) from competition among producers for the lowest cost of production, to competition between groups of producers who use sustainable practices versus those who do not, or between multiple certification schemes. This means that producers can do as a group what none of them can do individually: recoup the production costs of sustainable practices without being out-competed by less sustainable producers selling a cheaper product. This aligns individuals’ preferences with those of the group, and removes the social dilemma dynamic entirely.

Similarly, cultural transmission acts as certifications gain prestige, and the sustainability practice may be copied by other producers within or outside of the agricultural industry, as we note above. It is important to keep in mind that cultural transmission mechanisms are “selective” forces, in that individuals and firms strategically adopt behavior from others. Though much of the spread of certifications and natural labels is linked to individual consumer preferences, and therefore company profit, it is important to keep in mind that trait transmission must occur between groups as well, for example, through the emergence of new certification strategy for a unique product, or a large company adopting an in-house certified coffee label. A key point that affects selection for producers, consumers, and groups is that cheaters who produce or sell their products as sustainable but violate the rules of the relevant certification are punished, and this penalty shifts the cost of defection back to that individual producer.

The third mechanism, selective trait-based migration, is somewhat more subtle than the first two, because it does not directly change the global frequency of a given trait, but it simply reallocates traits between groups. As an example, consider a polluting industrial business which relocates to a state or country with weaker environmental regulations. This selective migration could result in more pollution overall, due to the less regulated context. When combined with cultural transmission, migration becomes more potent still: if the migrated business is successful, local businesses may imitate the strategy of gaining profits via the production of pollution externalities. Again, the mechanisms of cultural selection have the potential for larger effects when operating in concert.

A few caveats are important to keep in mind. Not every business success or case of social learning will produce an adaptive or functional practice, let alone a sustainable one. As in genetic evolution, selection mechanisms are not the only forces of cultural evolution: drift and sampling, for example, may limit cumulative culture adaptations (Kline and Boyd 2010), as will social network structure and population interconnectedness (Derex and Boyd 2016; Powell et al. 2009). Further, even when group-beneficial traits are the outcome of group-level cultural selection, it does not follow that the traits that the result will always be environmentally sustainable or ethically good. In fact, it merely means that these traits are “good” at self-reproduction. The traits spread, invade, and proliferate—much like a virus or internet meme, without regard for any society’s health, let alone its value system. For instance, in our certification example, it is only consumer demands and institutional enforcement that create a price premium for sustainably produced goods. Simply put, group-level cultural selection and the adaptations it creates are no panacea, and often contribute to sustainability crises rather than solving them. This is precisely why group-level cultural selection is important in sustainability: it can help to explain both progress and systemic failures.

## How to design a cultural multilevel evolutionary investigation

To design an explicit evolutionary investigation that includes both individual and group dynamics, researchers must choose focal components of a given social-ecological system to study. In practice, this is easiest when backed up with a solid ethnographic and/or historical understanding of the cultural and ecological system involved. Background work should include identifying candidate indicators or symptoms of group-level cultural adaptation: cooperative behaviors, enforcement behaviors, and institutional reinforcement (Fig. 1, Box 1). For example, in a small-scale fishery where sea turtle by-catch is causing significant ecological damage, a focal trait may be the voluntary use of turtle-exclusion devices on fishing nets by boat operators. Voluntary adoption of exclusion devices could be influenced by community recognition of declining sea turtle egg harvest on local beaches, and subsequent sanctions, or by a regional fisheries council attempting to gain certification for the whole fishery. In this way, the scale of interest could shift from individuals voluntarily adopting the practice to communities within the regional fishery. Many sustainability researchers will already have focal behaviors and institutions to study, but should still take care to define the focal cultural trait(s) in a way that facilitates tracking changes in frequency or intensity over time and/or across groups.

With focal traits selected, researchers must outline how a cultural trait fits into the group structure of the study system (Fig. 1, Box 2). Social structure and organization vary by population and context, so nested levels of social structure may include, for example, cities within states within nations, departments within colleges within universities within a university system, or nuclear families within villages within regional chiefdoms. Different types of groups, such as kinship groups and age cohorts, may be cross-cutting or overlapping with respect to membership. See Waring et al. (2015) for additional guidance in determining the relevant levels of social organization in a social-ecological system given a focal cultural trait. Because group structure is itself an object of study that can change over time, there is no need to empirically map the levels of social organization at this stage. Instead, researchers should treat social structure and organization as context for their hypotheses.

After defining their focal traits and the definition requires a group-level explanation, researchers need to construct an hypothesis detailing how the focal traits may function and spread (or not). Researchers can use the Fig. 1 flow chart to identify and describe traits that may be culturally evolved at the group level, and to propose the mechanisms—natural selection, cultural transmission, and/or migration—by which their focal trait may be shaped by cultural selection (Box 3). In using this tool, researchers should compare their interpretations against alternative explanations and contrary evidence to avoid just-so story telling. If the central hypothesis is that the focal trait is a group-level cultural adaptation, individual cultural evolution and decision-making should be considered as an alternative explanation.

Throughout this process, researchers should compile an evolutionary history of the focal trait. Though qualitative in nature, this step allows researchers to test their inferences about the possible environment in which the trait evolved, against historically documented facts. This also limits storytelling about the trait’s evolutionary history, and provides an historical line of reasoning to temper the purely adaptationist assumption that function is the only useful explanation for the form of a trait. There is no conflict between bringing together historical and functional explanations for a better understanding of a cultural trait. With the foundation for their study established, researchers should develop a plan to guide data collection. In designing a data collection protocol, it is useful to distinguish between mere indicators of a group-level cultural adaptation as discussed above, and direct empirical evidence that the conditions necessary for group-level cultural selection are fulfilled. If evidence supports the presence of these conditions, following Fig. 1, then the only conclusion is that group-level cultural selection is in play. This provides more conclusive evidence about the presence of selection, but it does not tell us whether the result is group-level cultural adaptation. Taken together, however, these two types of evidence create a strong case for group-level cultural selection resulting in a group-level cultural adaptation.

## How to tailor methods for data collection

Documenting both the indicators of group-level cultural adaptation as well as evidence for selection in action is ideal, but this sets a very high bar. To gather both types of evidence in full for a single case, researchers would likely need to collect detailed longitudinal data on cultural variation as well as material outcomes at both group and individual levels, over the time span during which a focal trait is under selection. Usually, such an extensive (and expensive) study is not feasible. However, sustainability researchers already have valuable qualitative and quantitative data, including histories and case studies that can be analyzed in a cultural evolutionary framework. The other articles in this special feature provide detailed examples. Because the spatiotemporal scales of cultural evolutionary forces are so variable, the best methods depend upon the context. In this section, we focus on three main criteria to aid researchers in study design: degree of control versus validity, time scale, and the complementary use of qualitative and quantitative data.

Our focus up to this point has been on observational studies, because these are the most immediately relevant for problems in sustainability and the creation of interventions. Observational studies have the advantage of validity in that they capture real-world behaviors, preferences, values, or other traits. In other words, these studies tell us what actually happens in the world. The price of this validity, however, is that observational studies lack experimental control, such that causal relationships can only be suggested by correlations, and the work cannot be directly replicated. Generally speaking, observational studies cannot tell us for sure which mechanisms or which conditions directly account for the outcomes we observe. Choosing how much control to trade off in the name of validity will depend on the research context and on the goals of the particular project, as with any research endeavor. The types of evidence we stipulate (see Sect. 4) provide a guide for researchers in navigating these tradeoffs.

For example, if the goal is to assess whether a group-level cultural adaptation exists in a given context, the researcher will naturally privilege validity and will use the indicators described above as a kind of list of symptoms by which to diagnose such an adaptation “in the wild”. This diagnostic approach could thus include identifying cooperative or enforcement behaviors in individuals and the presence of institutions supporting those behaviors. In contrast, if the research goal is to understand how these group-level culturally adapted institutions affect individual behavior, a better option is to prioritize control, in a laboratory or naturalistic experiment. Similar tradeoffs would also apply to gathering direct evidence for the conditions required for group-level cultural selection—where, for example, studies might range from totally uncontrolled historical analyses, to fully controlled mathematical models. Ideally, approaches on either end of the spectrum will be combined to tackle research problems from both ends. For example, the plausibility of historical narratives about how fisheries management systems in Fiji may function as group-level cultural adaptations (for discussion, see Waring et al. 2015) could be tested with mathematical or computational models that specify its social-ecological system much more rigorously than a verbal model can, and the model’s predictions could be tested against archeological and ecological data (e.g., Dean et al. 2000).

This example also highlights the issues of time scale and manner of sampling. Since evolution in general is an historical process, documenting the evidence of evolutionary change in its most direct form requires longitudinal data. However, group-level cultural selection, like biological evolution, is sometimes only observable over historical (or longer) timescales such that longitudinal data collection in the form of a pre-designed study may be impossible. In such cases, researchers can combine new and archival data on different time scales to address evidence for group-level cultural adaptations, and the three conditions for group-level cultural selection each in turn. For example, cross-sectional data describing waste use and littering behaviors at a single time point could give a snapshot of group-structured variation, and group-structured outcomes (for discussion, see Waring et al. 2015), while natural-experimental methods could be used to assess whether learning mechanisms shift these behaviors in that same population, treating transmission as a potential mechanism of spread. For context, an historical narrative might fill in the blanks with respect to how a potential transmission mechanism may act over time. Further, a mathematical model might offer a test of whether the verbal model’s assumptions lead to its purported outcomes in a cogent way.

Finally, in each of the examples above, we blend qualitative and quantitative data as well as analysis. We think both types of data are crucial in a multilevel analysis of cultural evolution, in particular because cultural evolution at the individual and group levels may often take place in different currencies. In our sustainability certification examples throughout, competition for maximizing profits may exist at the level of individual producers, but certification programs can link profit to consumer demands for sustainable practices due to culturally mediated prestige, social signaling, or other non-monetary currencies. Having a grasp on mixed methods means researchers can toggle between currencies, and also between qualitative and quantitative data, both of which can be analyzed either qualitatively or quantitatively (Bernard 2011). Qualitative ethnographic and/or historical knowledge is prerequisite to identify group-level cultural adaptations and to establish the social context within which a trait may have evolved. Similarly, quantitative data are necessary for evaluating the role and strength of selection, and formal mathematical models have the benefit of testing the plausibility of verbal arguments. Such models necessarily leave out qualitative detail, but can often teach us things that we did not expect to learn. Further, qualitative data and assessments can improve the quality of our quantitative data collection techniques—especially in cultural evolutionary research, where units of measure must be constructed to fit the context. Likewise, quantitative evidence can confirm or reject our qualitative interpretations, or merely put them into perspective and allow us to leverage observations to design better interventions. In any case, qualitative and quantitative data are reciprocally beneficial and we recommend a mixed methods approach incorporating both kinds of data and analysis (Bernard 2011).

## Assessing quantitative evidence

In cases where the appropriate quantitative data are available, it is possible to render estimates of group-level cultural selection mathematically. Two simple methods may be useful for researchers. The first approach is to use Rogers’ (1990) model of group selection by selective migration. Although the model is based on a genetic system driven by migration, its mechanistic assumptions are amenable to cultural group selection generally. Expression 1, below, presents Rogers inequality (1990, p. 401, Eq. 3) for the conditions under which an altruistic trait (individually costly, group beneficial) is favored by selection (also see Bowles 2006).

In this approach, two primary factors influence the likelihood of a cooperative trait or behavior to spread via group selection. The first factor, on the left of Expression 1, is the ratio of the average fitness cost to individuals within groups, *c*_w_, over the global fitness benefit of altruism across the whole population, *b*_p_. An altruistic trait is, by definition, relatively costly for individuals within groups, but beneficial to groups overall. So, when a trait confers greater net benefits for groups than net costs for individuals, the trait is more likely to evolve via group selection than individual selection. The second factor, on the right side, is the relative trait variation found between groups, versus between individuals. Simply put, if individuals within groups tend to share the same value of the trait, but groups differ significantly, the trait will influence group outcomes more than individual outcomes, and therefore be more likely to influence group evolution. The inequality (Expression 1) states that an altruistic trait will be favored by selection when the right side of the expression (the ratio of between-group variance, *F*_ST_, to within-group variance, *1* − *F*_st_) exceeds the left side (the ratio of within group costs, *c*_w_, to global net benefits, *b*_p_). The more the right side exceeds the left side, the more the trait will spread by group selection:

1 $$\frac{{c_{\text{w}} }}{{b_{\text{p}} }} < \frac{{F_{\text{st}} }}{{\left( {1 - F_{\text{st}} } \right)}}.$$

This method requires four types of quantitative data: individual traits, and group membership, and the costs and benefits of the trait for both individuals and groups. Most importantly, the dataset needs to include multiple groups and independent estimates of these values for each group. Provided appropriate data are available, employing Roger’s inequality is a simple process, but it only provides a true or false result. Usually, it would be better to compare the components of selection at the individual and group levels.

To compare the strengths of individual selection and group selection, we can use the Price equation (Okasha 2004; Price 1972). Doing so provides a richer metric than a true/false indication, using the same data. McElreath and Boyd’s (2007) formulation of Price equation,

2 $$\overline{w} \Delta \overline{z} = \underbrace {{\text{cov} (w_{g} ,z_{g} )}}_{{{\text{group}}\;{\text{selection}}}} + \underbrace {{E[\text{cov} (w_{ig} ,z_{ig} )]}}_{{{\text{individual}}\;{\text{selection}}}},$$

describes evolution, $\bar{w}\Delta \bar{z}$, as the product of the average change in trait frequency, $\Delta \bar{z}$, and average fitness, $\bar{w}$, and equates it with individual and group selection effects expressed in terms of the covariance between fitness and trait. In this way, individual selection is the covariance between individual traits, $z_{ig}$, and individual fitness within groups, $w_{ig}$, averaged across groups. While group selection is the covariance of group fitness, $w_{g}$, and group trait $z_{g}$. In a social dilemma, these two components hold opposite signs, so that their sum will reveal whether, overall, selection will favor the trait. This method for computing and comparing the components of selection is demonstrated in the cultural group selection simulations of Waring et al. (2017). Further methods can build upon these both approaches.

To help researchers use both techniques, and develop their intuitions for the mechanisms of group-structured cultural selection, we have provided an open-source tutorial written in R (R Development Core Team 2008), as an appendix (see ESM). The tutorial steps through the application of both expressions to a simulated group-structured data set. Scientists can alter the dataset, use their own data, and modify and build on the analytical code.

## Conclusion

We have described in detail how to design sustainability science research that takes advantage of the dynamics of cooperation and culture. Empirical sustainability research designed from the bottom up using this framework and predictions of cultural multilevel selection theory could shed new light on why and how sustainable practices emerge, persist, and disappear. With this, we offer a stepping stone to encourage explicit evolutionary research on the role of cultural evolution, including group-level cultural selection, on sustainability beliefs, practices, and institutions. Cultural evolutionary theory and the fundamentals for research design we provide here have potential to gain new traction for the field while building on existing research, theory, and methods rather than competing with already existing approaches.

## Electronic supplementary material

Below is the link to the electronic supplementary material.

Supplementary material 1 (PDF 226 kb)

Supplementary material 2 (DOCX 28 kb)

## Footnotes

1 Cultural group selection is a common term in the literature. Here, we use group-level cultural selection because we want to be clear that the competition between groups through group reproduction, group survival and group extinction is only one of many mechanisms which result in group-level cultural adaptations. For example, between-group cultural transmission may often play an important role in sustainability behaviors.
