# Supplementary material for: Designing cultural multilevel selection research for sustainability science
Source: Sustain Sci. 2017 Nov 21;13(1):9–19. doi: 10.1007/s11625-017-0509-2 (PMC6086275; doi:10.1007/s11625-017-0509-2)
Supplement: Supplementary file 1 — Supplementary material 1 (PDF 226 kb) [file 11625_2017_509_MOESM1_ESM.pdf]

Data analysis for cultural multilevel selection research: a tutorial

Tim Waring, timothy.waring@maine.edu

August, 2017

This R script is an analytical supplement to an article which describes how and why to use a cultural multilevel selection investigation to study the emergence of sustainability-related behaviors and cultural traits. The theory presented in the article, and the analysis we demonstrate below are not limited to environmental behavior, and may be applied to any appropriate behavioral dataset.

Here we provide a practical, reusable, and open-source analysis example for researchers interested in using cultural multilevel selection methods to analyize their data. If you use or build on the code, please cite:

> Designing cultural multilevel selection research for sustainability science. **Kline, Waring & Salerno**. (2018) *Sustainability Science*

Here we demonstrate two simple methods which will help researchers gain intuition about the statistical features of their datasets and whether group-level cultural selection may be at play. Because this code generates random data, running the code multiple times will produce different datasets, and different results.

Contents

Generate a group-structured dataset **2**

Using Rogers' Inequality **4**

Step 1: Estimate b, fitness benefit of altruism across whole population 4
Step 2: Estimate c, average fitness cost of altruism within groups 4
Step 3: Estimate Fst, proportion of trait variation due to groups 6
Step 4: Evaluate Roger's Inequality: *Is altruism favored by selection?* 6

Using the Price Equation **7**

Step 1: Calculate $Cov(W_g, z_g)$, group-level trait-fitness covariance 7
Step 2: Calculate $E(Cov(W_{ig}, z_{ig}))$, mean within-group trait-fitness covariance . . 8
Step 3: Compare $Cov(W_g, z_g)$ and $E(Cov(W_{ig}, z_{ig}))$ 8

Generate a group-structured dataset

It is always useful to create a simulated dataset so that you can practice and refine your analysis methods in advance of data collection and study design. We start by creating a simple, random, group structured dataset. You may change these values yourself by editing the code.

```
groups <- 8
indivs <- 20
```

Next, we generate a table of group-wise parameters which will be used to create the actual dataset. These include the presence of the trait under study, and its costs and benefits.

```
means <- data.frame(group=(1:groups),
                    trait_freq=round(runif(groups),2),
                    group_cost=sample(10,groups,replace=T),
                    group_benefit=sample(10,groups,replace=T),
                    group_sd=round(runif(groups, min=1, max=3),2))

means
```

```
##   group trait_freq group_cost group_benefit group_sd
## 1     1       0.49          5             9     2.64
## 2     2       0.62          8             9     1.83
## 3     3       0.83         10             8     2.26
## 4     4       0.51          7             9     1.72
## 5     5       0.46          4             6     1.89
## 6     6       0.16          3             3     1.31
## 7     7       0.78          1             8     2.70
## 8     8       0.24          9             5     1.86
```

Now we frame our simulated group-structured dataset.

```
group<-data.frame(group=(1:groups))
indiv<-data.frame(indiv=(1:indivs))
data<-merge(indiv,group)[,c(2,1)];rm(indiv,group)
data<-merge(data,means)
```

add individual level variation,

```
data$trait<-rbinom(indivs*groups,size=1,prob=data$trait_freq)
data$cost<-round(rnorm(indivs*groups,mean=data$group_cost,sd=data$group_sd),2)
data$benefit<-round(rnorm(indivs*groups,mean=data$group_benefit,sd=data$group_sd),2)
```

compute a simple random individual measure of fitness,

```
data$net <- data$benefit - data$cost
data<-data[,c("group","indiv","trait","net")]
```

and finish dataframe, the top five rows of which are:

```r
head(data)
```

```
##   group indiv trait  net
## 1     1     1     0 2.18
## 2     1     2     0 2.64
## 3     1     3     0 5.22
## 4     1     4     0 6.61
## 5     1     5     0 1.10
## 6     1     6     0 1.29
```

Using Rogers' Inequality

The first method we will demonstrate is applying Rogers (1990) inequality for evaluating whether, in a given case, group selection is strong enough to favor the evolution of an altruistic trait. Rogers' 1990 paper constructs a model of group selection by selective emigration, not on cultural diffusion or cultural selection. However, the assumptions he makes in his model are suitable for our purposes here. Rogers (1990), Page 401, equation 3 reads:

$$c/b < \frac{F_{st}}{(1 - F_{st})}$$

where:
b represents the fitness benefit of altruism across the entire population,
c denotes the average fitness cost of altruim within groups, and
F_{st} is the fraction of trait variation which can be attributed to groups. F_{st} is commonly used in population genetics, and is called the 'fixation index for population structure'.

Step 1: Estimate *b*, fitness benefit of altruism across whole population

To do this we calculate the average fitnesses of altruists and non-altruists and substract them.

```
altruist_fitness <- mean(data$net[data$trait == 1 ]) # mean fit of altruists
nonaltruist_fitness <- mean(data$net[data$trait == 0 ]) # mean fit of non-As
b <- altruist_fitness - nonaltruist_fitness

b
```

```
## [1] -0.02798246
```

Step 2: Estimate *c*, average fitness cost of altruism within groups

First, it is useful to make a group-wise data summary. This corresponds to our `means` dataframe, from above. We use `dplyr` to calculate group-wise means.

```
library(dplyr)
gstats<-as.data.frame(
    data[,c("group","trait","net")] %>%
        group_by(group) %>%
        summarise_all(mean))
```

```r
gstats
```

```
##   group trait     net
## 1     1  0.35  4.7465
## 2     2  0.65  1.2645
## 3     3  0.90 -1.9295
## 4     4  0.40  1.7640
## 5     5  0.25  2.4755
## 6     6  0.40 -0.0455
## 7     7  0.85  7.6410
## 8     8  0.40 -4.3035
```

Next, we calculate the fitness cost of altruism *within each group*.

```r
# mean fitness in groups by trait (altruist, non-altruist)
fit_gt <- data %>%
    group_by(group,trait) %>%
    summarize(fit = mean(net))

# pull mean groupwise fitness by trait into columns
fit_a <- fit_gt[fit_gt$trait == 1 ,c(1,3)]
fit_n <- fit_gt[fit_gt$trait == 0 ,c(1,3)]

# merge altruist fitness with groupwise dataframe, gstats
gstats<-left_join(gstats, fit_a, by="group")
gstats$fit_a <- gstats$fit
gstats$fit <- NULL

# merge non-altruist fitness with groupwise dataframe, gstats
gstats<-left_join(gstats, fit_n, by="group")
gstats$fit_n <- gstats$fit
gstats$fit <- NULL

# calculate fitness cost of altruism in each group
gstats$cost <- gstats$fit_n - gstats$fit_a
```

Finally, we take the mean across groups. In some cases there may be no altruists or no non-altruists in measured groups. In such cases, instead of using `NA`, a better assumption might be to set the fitness of the missing type to zero for that group. But for demonstration, `na.rm` will do.

```r
c<-mean(gstats$cost, na.rm=TRUE)
c
```

```
## [1] 0.3041318
```

Step 3: Estimate *Fst*, proportion of trait variation due to groups

For this we use the `hierfstat` package to calculate the components of variance. Make sure to specify `diploid=FALSE` since we are working with two mututally exclusive cultural types, analogous to two 'alleles' on a single 'gene.' More than just a simple F_{st}, the `varcomp` function provides information on population structure for any number of population groupings. Here we just extract a single F_{st} value for a single grouping on a single 'gene.'

```
library(hierfstat)

# Calculate Fst at group level, haploid system
Fst <- varcomp(data[,c("group","trait")], diploid=FALSE)$F
Fst <- as.numeric(Fst); Fst
```

```
## [1] 0.190396
```

Step 4: Evaluate Roger's Inequality: *Is altruism favored by selection?*

Now our estimates for each parameter in Roger's inequality:

```
b # fitness benefit of altruism across whole population
```

```
## [1] -0.02798246
```

```
c # average fitness cost of altruism within groups
```

```
## [1] 0.3041318
```

```
Fst # proportion of trait variation due to groups
```

```
## [1] 0.190396
```

Roger's inequality states that "*The altruistic allele is favored by selection when the ratio of between- to within-group variance exceeds the ratio of cost to benefit.*" Thus we can compute a simple binary test:

```
c/b < Fst/(1-Fst)
```

```
## [1] TRUE
```

But for demonstration purposes it is sufficient to show what types of data sets do show statistical signs of group selection, and which types do not. However, the simple thumbs-up, thumbs-down estimate may not be useful in many cases. For example, it would be nice to have a comparative measure of the strength of group and individual selection. The Price Equation provides such a better comparative metric, as we will demonstrate below.

Using the Price Equation

The Price Equation can be used to compare the relative effects of individual and group selection. We use McElreath and Boyd's (2007) formulation of Price equation, as follows:

$$\overline{W}\Delta\overline{Z} = Cov(W_g, z_g) + E(Cov(W_{ig}, z_{ig}))$$

The left hand side of the equation, $\overline{W}\Delta\overline{Z}$ denotes total change, or "evolution," where $\overline{W}$ is mean fitness, and $\Delta\overline{Z}$ is average change in trait. We will not compute these separately here, and they cannot be computed separately without temporal data.

On the right hand side we have:
W_g is the average fitness in group g,
Z_g is the trait frequency in group g,
W_{ig} is the fitness of individual i, in group g, and
W_{ig} is the trait of individual i, in group g, so that $Cov(W_g, z_g)$ is the covariance between group trait and group fitness, and
$E(Cov(W_{ig}, z_{ig})$ is average individual-level trait-fitness covariance.

Calculating and comparing these terms is very straightforward. We will use the same group-structued dataset, `data`, which we generated in the beginning. For clarity, some code will be redundant with the Rogers example.

Step 1: Calculate $Cov(W_g, z_g)$, group-level trait-fitness covariance

Do groups with the higher frequencies of the trait tend to have higher fitnesses?

```
library(dplyr)
gstats <-
    data %>%
    select(c("group","trait","net")) %>%
    group_by(group) %>%
    summarise_all(mean)

Group_TFC <- cov(gstats$net, gstats$trait)
Group_TFC
```

```
## [1] 0.1042214
```

Step 2: Calculate $E(Cov(W_{ig}, z_{ig}))$, mean within-group trait-fitness covariance

Now we calculate within-group trait-fitness covariances, and take their average. This is the same code as the previous step, with one new line. Note that in this example, groups are the same size.

```
gstats <-
    data %>%
    select(c("group","trait","net")) %>%
    group_by(group) %>%
    mutate(cov=cov(trait,net)) %>% # collect within-group covariances
    summarise_all(mean)
```

```
## Warning: package 'bindrcpp' was built under R version 3.3.2
```

```
Mean_Indiv_TFC <- mean(gstats$cov)
Mean_Indiv_TFC
```

```
## [1] -0.1033388
```

Step 3: Compare $Cov(W_g, z_g)$ and $E(Cov(W_{ig}, z_{ig}))$

Finally, we can compare the two trait-fitness terms, quantitatively. Here we will use a plot. The sum, `Total`, represents the total evolutionary change $\overline{W}\Delta\overline{Z}$. By comparing it with the individual and group-level trait-fitness covariances, we can see how they relate, and which is larger and therefore drives evolution overall.

```
Total <- Group_TFC + Mean_Indiv_TFC
plotdf <- data.frame(
    Level=c("Total", "Group", "Individual"),
    selection=c(Total,Group_TFC,Mean_Indiv_TFC))
```

Comparing the magnitude and sign of the different components of selection is the key portion of this exercise. This can be done by simply inspecting the estimates directly:

```
plotdf
```

```
##        Level     selection
## 1      Total  0.0008826128
## 2      Group  0.1042214286
## 3 Individual -0.1033388158
```

And it can also be visually represented. A basic barplot helps to show how individual and group-level selection interact to create total change.

```r
library(ggplot2)

ggplot(data=plotdf, aes(x=Level, y=selection)) +
    ylab("Strength of Selection\n (trait-fitness covariance)") +
    xlab("Level") +
    geom_bar(stat="identity") +
    scale_x_discrete(limits = c("Total", "Individual", "Group"))
```

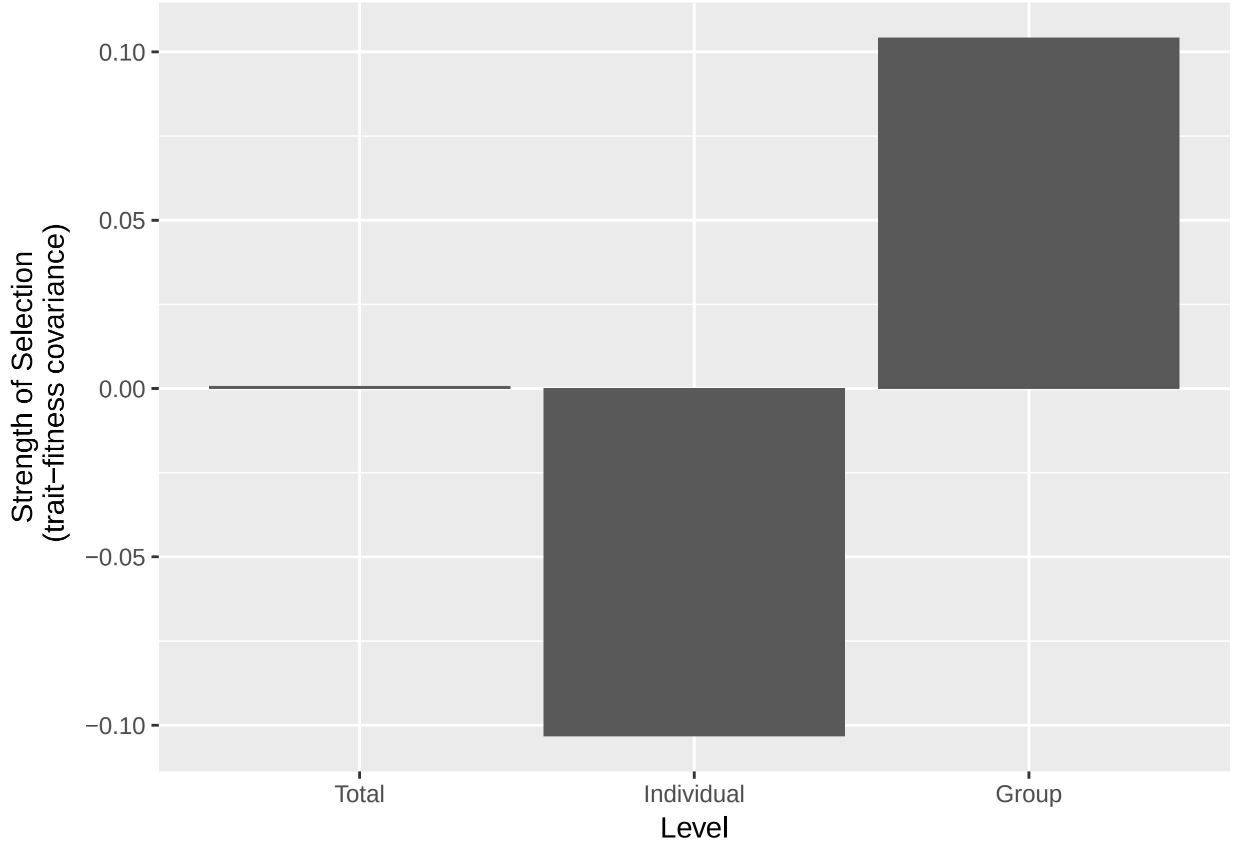

Two final points should be reiterated.

1. These data do not include change over time (multiple measurements of trait values), but these computations provide the basic approach which could be extended to time series data.
2. Also, because the dataset is randomly generated it may not produce interesting interactions between individual and group-level selection. We suggest running it a few times to see the various possibilities.

Tim Waring, 2017
University of Maine
