# Supplementary material for: Designing cultural multilevel selection research for sustainability science
Source: Sustain Sci. 2017 Nov 21;13(1):9–19. doi: 10.1007/s11625-017-0509-2 (PMC6086275; doi:10.1007/s11625-017-0509-2)
Supplement: Supplementary file 2 — Supplementary material 2 (DOCX 28 kb) [file 11625_2017_509_MOESM2_ESM.docx]

Waring, Timothy, 2017, "Data analysis for cultural multilevel selection research: a tutorial", [doi:10.7910/DVN/9HO5J8](http://dx.doi.org/10.7910/DVN/9HO5J8), Harvard Dataverse, V1
